# Antiplasmodial and Genotoxic Study of Selected Ghanaian Medicinal Plants

**DOI:** 10.1155/2020/1582724

**Published:** 2020-09-23

**Authors:** Selorme Adukpo, Doris Elewosi, Richard Harry Asmah, Alexander K. Nyarko, Patrick Kwaku Ekpe, Dominic Adotei Edoh, Michael Fokua Ofori

**Affiliations:** ^1^Noguchi Memorial Institute for Medical Research, University of Ghana, Legon, Ghana; ^2^School of Pharmacy, University of Ghana, Legon, Ghana; ^3^Department of Animal Biology and Conservation Science, University of Ghana, Legon, Ghana; ^4^University of Ghana School of Allied Health Sciences, Korle Bu, Ghana; ^5^University of Health and Allied Sciences, Ho, Ghana; ^6^Department of Plant and Environmental Biology, University of Ghana, Legon, Ghana

## Abstract

**Results:**

Five out of the eight plants, *A. boonei* stem bark, *S; siamea* Lam root, *M. lucida Benth* leaves, *P. niruri,* and *A. hispidum* DC whole plants, showed varying degrees of antiplasmodial activity against the asexual stage of the parasite. The most active extract against chloroquine-sensitive (3D7) and chloroquine-resistant (Dd2) *P. falciparum* strains is the *A. hispidum* extract which yielded a mean inhibitory concentration at 50% (IC_50_) of 3.66 *µ*g/ml and 3.71 *µ*g/ml for 3D7 and Dd2, respectively. This was followed by *S. siamea* Lam with 3.95 *µ*g/ml for 3D7 and 4.47 *µ*g/ml for Dd2. The IC_50_ values of the *A. boonei* extract against 3D7 and Dd2 *P. falciparum* parasites were 5.13 *µ*g/ml and 3.62 *µ*g/ml, respectively. For the *M. lucida Benth* extract, the least IC_50_ value was 6.46 *µ*g/ml. All five extracts exhibited dose-dependent antiplasmodial activity. Assessment of the genotoxic effects the *A. hispidum* extract by the comet assay revealed substantial damage to *P. falciparum* DNA.

**Conclusion:**

This study demonstrates that the crude extract of *A. hispidum* DC, one of the plants used traditionally to treat malaria, inhibits the growth of P. falciparum *in vitro* and could be a potential source of antimalarial drug. The report has highlighted genotoxic and cytotoxic effects of the selected plant extracts on human leukocytes as well.

## 1. Introduction

Malaria in humans is caused by five species of haemospozoan parasites, namely, *Plasmodium falciparum*, *P. malariae*, *P. ovalae*, and *P. vivax* and, lately, the Zoonotic *P. knowlesi* [1]. Of these, *P. falciparum* is the most widespread and causes the most lethal infection with the female mosquito of the *Anopheles gambiae complex* being responsible for the transmission of the disease [1]. The disease threatens billions of people globally. Infection with the parasite may result in asymptomatic or clinical malaria [1] with about 2% of the clinical cases resulting in severe disease [2] that accounts for thousands of deaths attributed annually to malaria [1]. More than 90% of these deaths occur in sub-Saharan Africa of which children are in the majority. Mortality in children under five years of age is quite high, but the disease affects all age groups, including pregnant women and nonimmune adults either residing in or visiting endemic areas. Low birth weight, spontaneous abortion, and stillbirth that are associated with placental malaria are some of the other effects of malaria on humans [1].

To control the global malaria havoc, preventive and curative measures were initiated in the 1950s to eradicate the disease following the discoveries of the causative parasite, mode of transmission of the parasite, the vector, and antimalaria drugs. Although some successes were chalked by the campaign in wiping out the disease in the temperate regions, it failed in tropical countries and was abandoned in 1969. The disease still lingers on, and infection is on the increase probably due to the emergence of resistance of the parasite to most of the common and affordable drugs such as chloroquine [1].

Until recently, chloroquine was the first-line drug for treating and preventing malaria in Ghana and many other countries. However, there is widespread of *P. falciparum* in circulation that is resistant to chloroquine and other drugs such as sulfadoxine-pyrimethamine and proguanil [3]. The worsening resistance [4, 5] has resulted in a change from chloroquine to artemisinin-based combination therapies such as Coaterm, but these are very expensive and, hence, not readily accessible to most of the rural folks. This, coupled with a lack of an efficacious malaria vaccine, has necessitated the search for novel, well-tolerated, newer, cheaper, accessible, more efficient, and safer antimalarial drugs [6].

Natural products are important sources of biologically active compounds that could be potential sources of novel antimalarial drugs. The WHO estimates that 80% of the world population use herbal medicine for some aspect of primary health care [7]. Herbal medicine is, thus, a major component of all indigenous peoples' traditional medicine and is a common element in homeopathic, naturopathic, traditional healing, and native medicine [8].

In Ghana and some other African countries, herbal medicinal preparations from plants such as *Triclisia* leaves, *Jatropha spp, M. lucida* Benth*, Cryptolepis sanguinolenta* (Lindl) Schlt*, Khaya senegalensis,* and *Azadirachta indica* are commonly used either alone or in combination with other plants for the treatment of fevers and other conditions such as malaria [9–15]. While some medicinal plants have shown a great deal of antimalarial activities, either as schizontocidal and/or prophylactic agents [16, 17], others have not been investigated scientifically. In this study, we report on the efficacy, cytotoxicity, and genotoxicity of extracts from eight medicinal plants that have been reported to possess antimalarial properties or used by traditional healers in the treatment of malaria [9, 11, 12, 15, 18].

## 2. Methodology

### 2.1. Collection of Plants

The root of *Senna siamea* Lam and *Cocos nucifera* L, the stem bark of *Alstonia boonei* (De Wild), leaves *Morinda lucida* Benth and of *Psidium guajava* Linn, and whole plant of *Phyllanthus niruri* L*, Acanthospermum hispidum* DC, and *Cymbopogon citratus* (DC.) Stapf were collected between June and July 2008 from the University of Ghana's botanical garden at Legon. Each plant was identified with the help of Mr Patrick P. Ekpe, a Taxonomist at the Department of Plant and Environmental Biology, University of Ghana, Legon. The plants, *Acanthospermum hispidum* DC (DEDE/2008/001), *Alstonia boonei* (DEDE/2008/002), *Cocos nucifera* L (DEDE/2008/003), *Cymbopogon citratus* (DC.) Stapf (DEDE/2008/004), *Morinda lucida* Benth (DEDE/2008/005), *Phyllanthus niruri* L (DEDE/2008/006), *Psidium guajava* (DEDE/2008/007), and *Senna siamea* Lam (DEDE/2008/008), were collected and assigned collection numbers as indicated in the parentheses mentioned above. The voucher specimen of each plant was prepared and deposited in the Ghana Herbarium at the Department of Plant and Environmental Biology, University of Ghana, Legon, Ghana.

#### 2.1.1. Preparation of the Crude Extracts

The parts of *A. boonei*, *S. siamea* Lam, *M. lucida* Benth, *P. guajava*, *P. niruri* L, *A. hispidum* DC, and *Cymbopogon citratus* (DC.) Stapf were washed thoroughly with potable water, followed by distilled water, and air-dried at room temperature. For standardization purposes, 200 g of each sample was weighed and boiled in 800 ml distilled water for an hour to obtain the extracts. The aqueous extracts were collected and centrifuged twice at 2000 rpm for 10 minutes to remove particulate materials. Each of the supernatants was, then, freeze-dried in a Hepto Power Dry LL3000 machine (Jouan Nordic, Denmark) and stored in a dedicated refrigerator until later use. Twenty milligrams (20 mg) of each of the freeze-dried plant extract was reconstituted in one millilitre of sterile distilled water (1 ml) to obtain a concentration of 20 mg/ml. Each of the solutions was filtered successively through membranes of 0.45 *µ*m pores and 0.22 *µ*m pores (Millipore Corp., Bedford) in a laminar flow biosafety cabinet. Two-fold serial dilutions of each of the plant extracts were prepared to start from 833.3 *µ*g/ml to 6.5 *µ*g/ml in a parasite culture medium (RPMI 1640, L-glutamine, Gentamycin, and Albumax). All dilutions, including that of artesunate which was used as the positive control, were prepared fresh on the day of the assays.

#### 2.1.2. Growth Inhibition, Cytotoxicity, and Genotoxicity Assays

Parasites were cultured in O^+^ and sickle-negative human blood and coincubated with different concentrations of the various extracts and growth inhibition determined as described earlier [19]. The cytotoxic and genotoxic effects of the extracts on both parasites and human peripheral mononuclear cells (PBMCs) were evaluated by coculturing the parasites with the extracts at the predetermined concentrations. The cytotoxic effect of the extract on the PBMCs was assessed by the colorimetric determination of human lactate dehydrogenase released into the supernatant of the culture media [20]. The genotoxic effect of the extracts on either the parasites or the PBMCs was evaluated by the comet assay [21] using a commercially available Comet assay kit (4250-050-K, Trevigen, USA). The parasite DNA was stained with silver stain, while the DNA of the PBMCs was dyed with Sybr green.

#### 2.1.3. Statistical Analysis

GraphPad Prism version 8.4 (GraphPad Software, San Diego, CA, USA) and Microsoft Excel software were used to analyze the data and plot graphs. Mean values of repeated experiments presented as percentage growth inhibition of the various extracts were evaluated with the 50% growth inhibitory concentration (IC_50_) for the various extract calculated by a nonlinear regression analysis. Differences among three or more groups were assessed with ANOVA followed by Turkey post hoc correction analyses, while differences between any two groups were evaluated by Student's *T*-test analysis at *p* < 0.05.

## 3. Results

Two different laboratory strains of the *P. falciparum* parasite (3D7 which is chloroquine-sensitive and Dd2, chloroquine-resistant strain) were successfully cultured and used to evaluate the *in vitro* efficacy of seven selected medicinal plant extracts. All the extracts were screened with the parasites, but three did not show any appreciable antiplasmodial activities and were dropped from further *in vitro* analyses.

### 3.1. Effect of Extracts on the Growth of *Plasmodium falciparum* 3D7 and Dd2 Strains

**Figure 1:**
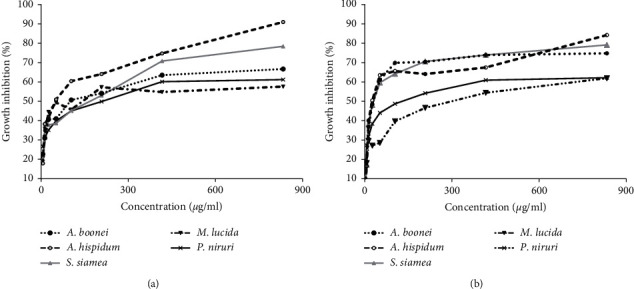
Concentration-dependent *Plasmodium falciparum* growth inhibition by various extracts. The top panel (a) shows the growth inhibitory characteristics of *Alstonia boonei, Senna siamea* Lam, and *Acanthospermum hispadum* of chloroquine-sensitive (3D7) strain of *Plasmodium falciparum*. The lower panel (b) represents the growth inhibitory activity of the various extracts on chloroquine-resistant (Dd2) strains of *P. falciparum*.

The inhibitory characteristics of the various extracts and the standard drug, artesunate, on the growth of the two strains of *P. falciparum* are presented in Figure 1 with the IC_50_ presented in Table 1. The least concentration 6.51 *µ*g/ml of each extract significantly inhibited the growth of either strain of *P. falciparum* used compared to the control (*p* < 0.001). The *P. falciparum* growth inhibition by *A. boonei* was concentration-dependent which ranges from 18.86% to 71.67% (Figure 1(a)). The highest concentration of 833.3 *µ*g/ml inhibited the growth of the 3D7 parasite up to 71.67%, while in the case of Dd2, the percentage growth inhibition reached a maximum of 66.67%. In the case of the *S. siamea* Lam extract, the percentage growth inhibition of the 3D7 strain of *P. falciparum* ranges from 17.29% for the minimum 6.5 *µ*g/ml to 79.11% (for a maximum of 833.3 *µ*g/ml concentration used, respectively) (Figure 1(b)). The *Morinda lucida* Benth extract inhibited the growth of both strains of *P. falciparum* parasites used (Figures 1(a) and 1(b)). The least concentration of 6.5 *µ*g/ml inhibited the growth of 3D7 up to 18.06% with the maximum concentration of 833.3 *µ*g/ml inhibiting the parasite growth up to 61.94% (Figures 1(a)). Similarly, the same minimum and maximum concentration of the extract inhibited the growth of Dd2 from 20.63% to 57.59% (Figures 1(b)). The *Phyllanthus niruri* L extract also inhibited the growth of both strains of *P. falciparum* (Figures 1(a) and 1(b)). The growth inhibition ranges from 27.29% to 62.19%. The most concentrated preparation at 833.3 *µ*g/ml inhibited the growth of chloroquine-sensitive parasite strain (3D7) up to 62.19%, and in the case of chloroquine-resistant strain (Dd2), the percentage growth inhibition was about the same (Figures 1(a) and 1(b)). Similarly, the *A. hispidum* DC extract showed growth inhibition of chloroquine-sensitive strain of *P. falciparum* ranging from 17.91% at 6.5 *µ*g/ml to 90.95% at 833.3 *µ*g/ml for the maximum concentration, whiles in the case of chloroquine-resistant strain, the minimum and maximum percentage growth inhibition were 16.61% and 84.22%, respectively (Figure 1). In general, the degree of *P. falciparum* strain 3D7 growth inhibition increased significantly with increasing extract concentrations up to 26.042 *µ*g/ml (*p*=0.032) for *A. boonei* and (*p*=0.046) for *S. siamea* but to only 13.021 *µ*g/ml in the case of *A. hispidum* (*p*=0.003) and *M. lucida* Benth (*p*=0.021). The rest, including data on Dd2, increased gradually and never reached significant levels (Figure 1). There was no significant difference amongst the IC_50_ when the IC_50_ values obtained for all the extracts for 3D7 (*p*=0.45) or Dd2 (*p*=0.11) were compared. Similarly, no differences were detected between the IC_50_ for the two *Plasmodium falciparum* strains (Table 1).

**Figure 2:**
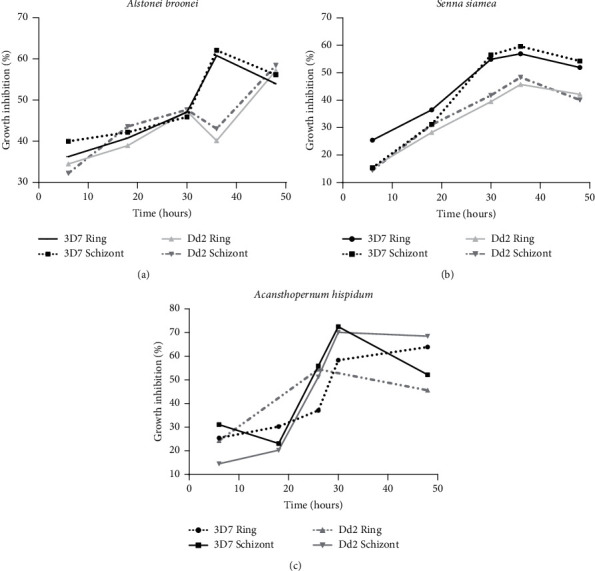
Concentration-dependent parasite stage-specific inhibition kinetics. The graphs show both ring and schizont stage-specific inhibitions mediated by *Alstonia boonei* (a), *Senna siamea* Lam (b), and *Acanthospermum hispidum* (c).

The percentage growth inhibition of ring and schizont developmental stages of 3D7 strain of the *Plasmodium* parasite with time is represented in Figure 2. The percentage growth inhibition of ring developmental stage by the *A. hispidum* DC extract increased gradually from the 6^th^ (21.41%) to the 48^th^ hour (63.88%) when the assay was terminated (Figure 2(c)). Growth inhibition leveled off from 18 hours to 24 hours, followed by an increase to 67.47% at 36 hours. It, then, dropped sharply to 52.12% at 48 hours. There was no significant difference between the growth inhibitory effects of *A. boonei* (De Wild) and *S. siamea* Lam extracts on the rings and schizont developmental stages of the malaria parasites (Figure 2). With the chloroquine-resistant strains (Dd2), growth inhibition by *A. boonei* (De Wild), *S. siamea* Lam, and *A. hispidum* DC extracts increased with time (Figures 2(a)–2(c)). The increase in growth inhibition for the developmental stages was from the 6^th^ to the 36^th^ hour. Growth inhibition, then, dropped from 44.11% to 41.13% for *S. siamea* Lam extracts and from 58.71% to 45.57% for *A. hispidum* DC. There was, however, a gradual increase of growth inhibition for the *A. boonei* (De Wild) extract from the 36^th^ to the 48^th^ hour when the assay was terminated (Figure 2).

### 3.2. Effect of Extracts on *Plasmodium* DNA

**Figure 3:**
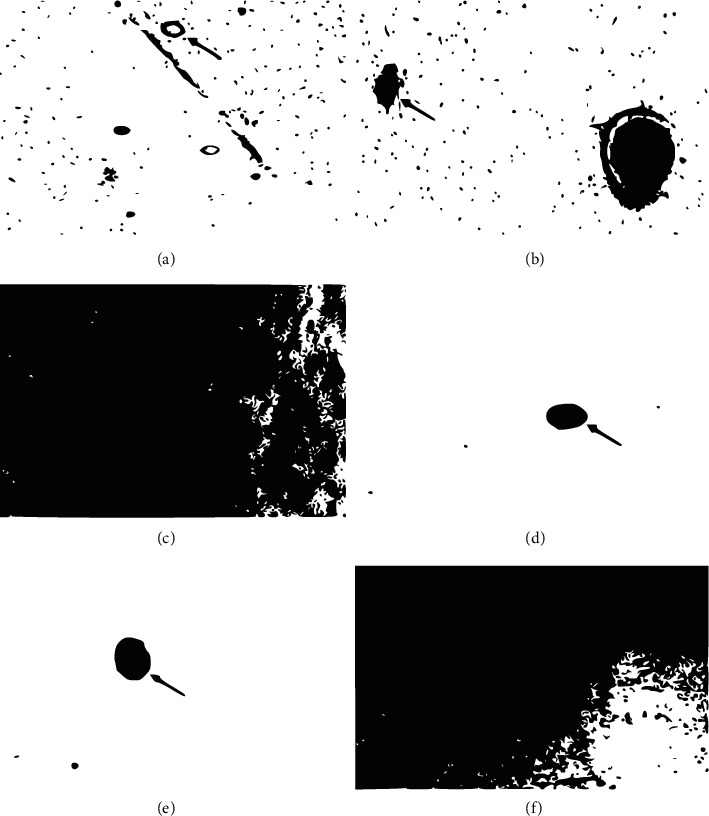
Photomicrographs of infected RBC and uninfected RBCs treated with the *A. hispidum*. extract. The figure shows photomicrographs of uninfected RBCs (a), infected RBCs without treatment (b), infected RBCs treated with 13.0 *µ*g/ml (c), infected RBCs treated with 52.1 *µ*g/ml (d), infected RBCs treated with 104.1 *µ*g/ml (e), and infected RBCs treated with 416.6 *µ*g/ml (f). Arrows show DNA comets of the parasite.

The effect of the plant extracts on *P. falciparum* DNA is presented in Figures 3. Undamaged cells appeared as intact nuclei without tails (negative control Figure 3(a)), whereas damaged cells had different appearances (B–F), with some appearing as comet (Figure 3(f)), unwinding, or distortion of cells DNA. A visual scoring and quantification of the extent of DNA damage produced an average score of 1.5 for the lowest diluted extract corresponding to 42% DNA in the tail of the comet. The highest score of 4 which translated to 75% DNA in the comet tail was recorded for the highest concentration of the *A. hispidum* DC extract (Table 2). The *Acanthospermum hispidum* DC extract also had a comet score of 4 (75% of DNA in the tail of the comet). The *Senna siamea* Lam extract, on the hand, had a score of 3 (corresponding to 65% of parasite DNA in comet tail (Table 2).

Visual examination and scoring of the comet were carried out according to the guidelines provided by the manufacturer [22, 23]. The results are presented as average comet score and average percentage scores. The higher the score or percentage score is, the more destructive the extract is to the DNA.

### 3.3. Cytotoxicity of the Extracts to Human Peripheral Blood Mononuclear Cells

Cytotoxicity of the three most active extracts determined by LDH leakages from the human peripheral blood mononuclear cells (PBMCs) is presented in Table 3. The table shows that *A. boonei* (De Wild), *S. siamea* Lam, and *A. hispidum* DC extracts-mediated hLDH release was concentration-dependent (Table 3). Comparing the three extracts, *A. hispidum* DC seems to cause more LDH leakage. The *Alstonia boonei* (De Wild) extract caused the least release of LDH (Table 3). Based on the percentage LDH leakage, IC_50_ was determined using the regression curve. The IC_50_ values estimated were 7.58 *μ*g/ml, 7.65 *μ*g/ml, and 3.46 *μ*g/ml for extracts *A. boonei* (De Wild), *S. siamea* Lam, and *A. hispidum* DC, respectively. All the three extracts were active and selectively toxic to the parasites than mammalian cells with selective toxicity indices ranging from 93 to 209 (Table 4).

The table shows the percentage of leakage of human lactate dehydrogenase from human peripheral mononuclear cells when the cells were subjected to various concentrations of the extracts. The greater the percentage leakage, the greater the cytotoxicity of the extract at the concentration.

#### 3.3.1. Qualitative and Quantitative Interpretation of Results

**Figure 4:**
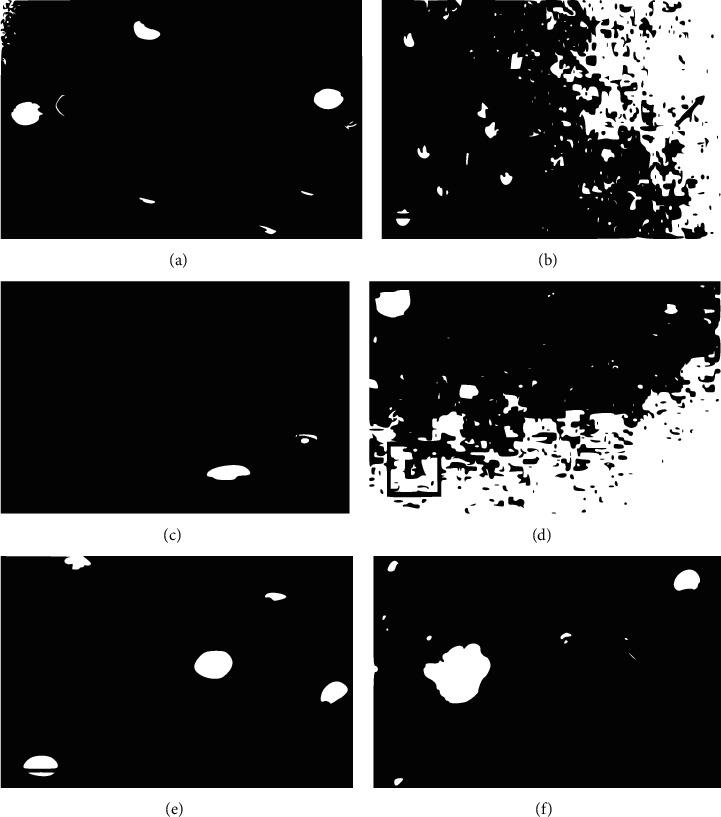
Photomicrographs of the effect of *A. hispidum* extract on the DNA of peripheral mononuclear cells (PBMC). The arrows point to cells. The bigger the cell and leaky the DNA from the nucleus, the more glowing it is. (a) The experimental arm without treatment, (b) was PBMC treated with 3.25 *µ*g/ml, (c) was treated with 13.01 *µ*g/ml, (d) was treated with 52.1 *µ*g/ml, (e) was treated with 208.1, and (f) treated with 833.3 *µ*g/ml.

The results of exposure of PBMCs to extracts are presented in (Figure 4). Undamaged cells have their nuclei intact without comet tails or diffused DNA as in the negative control panel (Figure 4(a)), whereas damaged cells have different appearances (B–F). This includes unwinding or distortion of DNA in the cells. *Alstonia boonei* (De Wild)*, S. siamea* Lam, and *A. hispidum* DC extracts had the highest comet score of 3, and the percentage DNA in the comet tail seen was 75% for *A. hispidum* and 70% for *S. siamea* Lam of DNA in the comet tail (Table 5). Extract *A. boonei* (De Wild) recorded 65% DNA in the comet tail.

Visual examination and scoring of the comet were carried out according to a set of guidelines [22, 23].

## 4. Discussion

The widespread resistance of the *Plasmodium falciparum* parasite to drugs being used to treat malaria poses a lot of public health challenges. Should the *P. falciparum* develop resistance to artemisinin, the last surviving most potent antimalarial drug, the havoc will be unprecedented. There is, therefore, an urgent need for novel and affordable drugs to either standalone or partner artemisinin for management of malaria; hence, this investigation into selected medicinal herbs that are used in treating malaria in Ghana and some other countries in Africa [9, 11–15, 24, 25]. Aqueous extracts from all the plants tested showed various degrees of antiplasmodial activities against both chloroquine-sensitive 3D7 and chloroquine-resistant Dd2 laboratory strains of *P. falciparum*. The observed sensitivity of both parasite lines to the extract lends more credence to their use traditionally as antimalarial plants. From rankings of the IC_50_s of the extracts, the *A. hispidum* extract was the most efficacious, closely followed by *A. boonei* (De Wild) and *S. siamea* Lam. They inhibited parasite growth in a concentration-dependent manner, and this seems to result from tempering of the integrity of *P. falciparum* parasite DNA by the plant extracts. This observation is consistent with other studies that have demonstrated that herbal preparations [26, 27], as well as chloroquine [28] disrupt *P. falciparum* parasite DNA.

*Alstonia boonei* (De Wild) displayed high activity against chloroquine-resistant *P. falciparum* parasites. This confirms earlier *in vitro* and *in vivo* [29] reports which suggested the plant as a source of potentially useful lead substances for future antimalarial drugs. Currently, artemisinin, a plant-derived drug is the most potent drug against chloroquine-resistant parasites [30]. To minimize the development of resistance by *P. falciparum* parasites to artemisinin, artemisinin combination therapy has been recommended by the World Health Organization. The exact-active antimalarial agent in *A. boonei* (De Wild) could, therefore, be identified and developed into an antimalarial drug that may be used as a standalone or be in combination with artemisinin to treat malaria.

The *Senna siamea* Lam extract displayed high antiplasmodial activity in this study. This corroborates reports of the use of the plant in countries in Central and West Africa, including Ghana for the management of malaria [31–33]. Interestingly, the observed IC_50_ value of 4.48 *µ*g/ml is about five-folds lower than what was reported by Sanon et al. [34]. This might have arisen from the plant part used and the type of solvent used, as well as the duration of extraction of the active agent relative to other products during the preparation of the extract. The aqueous extraction employed in the current report was made from the root of the plant by boiling at 100°C for 60 minutes. However, Sanon et al. made their aqueous extraction from the leaves of the plant and the duration of extraction was shorter [34]. It is, thus, possible that the active antiplasmodial ingredient in the plant was more concentrated in the root bark. These notwithstanding, it is very clear that extracts from either part of *Senna siamea* Lam have antiplasmodial activity that can be isolated and developed into a novel, a readily available, and affordable antimalaria drug.

*Morinda lucida Benth* and *P. niruri* L extracts separately showed relatively low antiplasmodial activity *in vitro.* Even though this is in agreement with an earlier report [33], it contrasts sharply with others [12, 33]. The difference in the current and the earlier reports might be due to the solvent used in the extraction as it has been well documented that plants might show no activity against *Plasmodium* species when extracted with polar solvents but exhibit extensive antimalarial/antiplasmodial activity when they are extracted with organic solvents such as ether or chloroform [32, 33]. This situation may be due to the polarity of the bioactive agent and, hence, its solubility in solvents with varying polarities. The current data, together with that from Chithambo and colleagues [12], therefore, suggest that the main antiplasmodial agent in these plants might be more soluble in organic solvents than polar ones such as water. Comparatively, the *A. hispidum* extract showed a more effective growth inhibitory effect on the two strains of *P. falciparum* parasites than the rest of the extracts. The high level of antiplasmodial activity of this extract against both strains of *P. falciparum* agrees with earlier findings [32, 33] by Sanon and his colleagues [34].

Extracts of *C. Nucifera* L*, P. guajava*, and *C. citratus*, which were reported as useful for the management of malaria [10, 18, 35, 36], did not demonstrate any appreciable antiplasmodial activity in this study. These studies were performed *in vitro* and it is possible that the constituents in the plants might require bioactivation *in vivo* to exhibit antiplasmodial activity [12].

Assessment of the extracts by the comet assay to evaluate their genotoxic effects suggested that the plants act via damaging parasite DNA. This is akin to the mechanism by which chloroquine inhibits parasite growth [30], although the selective indices of these plants are not as high as values reported earlier for chloroquine. This is not surprising because whereas chloroquine is a pure substance, the plant materials used are total extracts, which included phytoconstituents that may not have antiplasmodial activity. The genotoxic effects observed are in line with an earlier report that *A. hispidum* is toxic in both *in vitro* and *in vivo* [37], which extends to its antimalarial or antimicrobial activity as per these and other findings [38].

It is not clear which phytoconstituents are responsible for the antiplasmodial activities observed in these studies. However, phytochemical studies have shown that alkaloids, saponins tannins, flavonoids, steroids and glycosides, terpenes, anthraquinones, and glycoside abound in the plants under study [39, 40] which have been suggested to possess varying degrees of antimalarial activities depending upon extraction media and parasite strains/isolates used [39–41]. It is, therefore, reasonable to postulate that these phytoconstituents, either singly or in combinations may be responsible for the observed antiplasmodial activities recorded.

In conclusion, the *Alstonia boonei*, *Senna siamea* Lam, and *Acanthospermum hispidum* extract showed good antiplasmodial activity which warrants further investigations to ascertain their usefulness as good antimalarial agents. It will, therefore, be useful to carry out activity-guided fractionation studies to identity the antiplasmodial active phytoconstituents in the extracts for further development.

## Figures and Tables

**Table 1:** IC_50_ of extracts on the growth of two laboratory strains of *Plasmodium falciparum*.

EXTRACT	Mean IC_50_ (*µ*g/ml) of extracts on Dd2	Mean IC_50_ (*µ*g/ml) of extracts on 3D7	*p* value
*Acanthospermum hispidium* DC	3.70 ± 0.08	3.66 ± 0.06	0.70
*Alstonia boonei* (De Wild)	3.62 ± 0.20	5.47 ± 0.02	0.79
*Senna siamea* Lam	4.47 ± 0.11	3.95 ± 0.06	0.13
*Murinda lucida* Benth	6.47 ± 0.12	5.50 ± 0.06	0.52
*Phyllanthus niruri* L	5.74 ± 0.20	5.47 ± 0.02	0.57
Artesunate	0.006 ± 0.0004	0.006 ± 0.0003	0.85

The table shows the IC_50_ of the various extracts. The results are presented as mean ± 2 standard deviations.

**Table 2:** Concentration-dependent genotoxicity of the extract/drug to the parasite.

Extract/drug (concentration, *µ*g/ml)	Comet score scale	Average (%) comet score
Control	0	0
Infected red blood cells	2	46
*A. hispidum* DC (6.5)	1.5	42
*A. hispidum* DC (52.1)	2	46
*A. hispidum* DC (208.3)	3	65.5
*A. hispidum* DC (833.3)	4	75
*Alstonia boonei* (6.5)	1.5	42
*Alstonia boonei* (52.1)	2	62
*Alstonia boonei* (208.3)	3	65
*Alstonia boonei* (833.3)	3	65
*Senna siamea* Lam (6.5)	1.5	42
*Senna siamea* Lam (52.1)	2	56.5
*Senna siamea* Lam (208.3)	2	65
*Senna siamea* Lam (833.3)	3	65
Artesuanate (0.006)	3	85

**Table 3:** Dose-dependent LDH leakages from human peripheral mononuclear cells.

Extract concentration (*µ*g/ml)	Lactate dehydrogenase (LDH) leakage (%)		
	*Alstonia boonei*	*S. siamea* Lam	*A. hispidum* DC
3.2	21.17	22.36	24.20
13.02	21.46	22.75	25.83
52.10	21.86	23.17	27.45
208.33	23.59	23.86	34.45
833.33	40.87	45.84	78.94

**Table 4:** Mean IC_50_ of cytotoxicity values and selective indices of the three most active extracts.

Extracts	Mean IC_50_ (*µ*g/ml) of extracts on PBMCs	Selective index (SI^a^) of extracts on Dd2 (%)	Selective index (SI^a^) of extracts on 3D7 (%)
*A. hispidium* DC	3.46 ± 0.25	93.33	94.40
*Alstonia boonei*	7.58 ± 0.21	209.42	147.78
*Senna siamea* Lam	7.65 ± 0.22	171.05	193.57

Selective indices (SI^a^) = cytotoxicity IC_50_/antiplasmodial IC_50_ × 100. The IC_50_ results are presented as mean ± 2 standard deviations.

**Table 5:** Genotoxic effect of the extracts on human peripheral mononuclear cells (PBMCs).

Extract added (concentration, *µ*g/ml)	Visual comet score (data set 1)	Visual comet score (data set 2)	Comet score (average)	% DNA in tail of comet
Control	0	0	0	0
*A. hispidum* (3.3)	2	2	1.5	20
*A. hispidum* (13.0)	2	2	2	30
*A. hispidum* (52.1)	2	2	2	45
*A. hispidum* (208.3)	2	2	2	55
*A. hispidum* (833.3)	3	3	3	75
*Alstonia boonei* (3.3)	2	2	1.5	20
*Alstonia boonei* (13.0)	2	2	2	25
*Alstonia boonei* (52.1)	2	2	2	30
*Alstonia boonei* (208.3)	2	2	2	46
*Alstonia boonei* (833.3)	3	3	3	65
*Senna siamea* Lam (3.3)	2	2	1.5	20
*Senna siamea* Lam (13.0)	2	2	2	35
*Senna simea* Lam (52.1)	2	2	2	40
*Senna siamea* Lam (208.3)	2	2	2	55
*Senna siamea* Lam (833.3)	3	3	3	70

## Data Availability

The data used to support the findings of this study are included within the article.
